# Physical and Chemical Effects of Different Working Gases in Coffee Brewing: A Case Study of Caffè Firenze

**DOI:** 10.3390/foods9121825

**Published:** 2020-12-09

**Authors:** Giulia Angeloni, Piernicola Masella, Lorenzo Guerrini, Agnese Spadi, Maria Bellumori, Marzia Innocenti, Alessandro Parenti

**Affiliations:** 1 DAGRI, Department of Agriculture, Food, Environment and Forestry, University of Florence, Piazzale delle Cascine, 16, 50144 Firenze, Italy; piernicola.masella@unifi.it (P.M.); lorenzo.guerrini@unifi.it (L.G.); agnese.spadi@unifi.it (A.S.); alessandro.parenti@unifi.it (A.P.); 2 Department of Neurofarba, Division of Pharmaceutical and Nutraceutical Sciences, University of Florence, via Ugo Schiff, 6, 50137 Sesto Fiorentino, Italy; maria.bellumori@unifi.it (M.B.); marzia.innocenti@unifi.it (M.I.)

**Keywords:** espresso foam, extraction, foam index, foam persistence, caffeine

## Abstract

(1) Background: Recently, a new espresso extraction method, *Caffè Firenze,* has been developed, which uses gas at operating pressures of 20 bar to obtain abundant, persistent foam. The experiment aimed to evaluate the effect of using six gases (air, argon, nitrogen, carbon dioxide, carbon/nitrogen mix, and nitrous oxide) on the foam and liquid coffee. (2) Methods: Foam volume, persistence, sugar retention time, color, and rheological properties were measured. Volatile organic compounds were also evaluated. Analyses were also carried out on the liquid coffee to determine caffeine and chlorogenic acid concentrations. (3) Results: The analysis of variance revealed significant differences between the gases for all parameters. Multivariate analysis identified three groups of gases: the first comprised air, N_2_, and Ar; the second CO_2_ and N_2_O; and the third comprised samples extracted with CO_2_/N_2_ mix. (4) Conclusions: The choice of gas significantly influences the drink’s chemical-physical characteristics and is fundamental for product diversification.

## 1. Introduction

Coffee is one of the world’s principal commodities. It is also one of the most widely-consumed beverages as a result of its pleasant taste and aroma, not to mention its stimulant qualities. Numerous brewing apparatuses have been developed, and it is known that different brewing techniques produce beverages of different quality [1,2]. One example is espresso coffee, which is prepared with hot water (90 ± 5 °C) at high pressure (9 bar) that percolates for a short period (30 ± 5 s) through a layer of roasted, ground beans (6.5 ± 1.5 g).

Foam is the result of the coarse dispersion of gas bubbles in a continuous liquid phase [3]. In espresso, the gas phase mainly consists of carbon dioxide generated during roasting and trapped within the cell structure. The continuous phase is an oil in water emulsion of microscopic droplets (90% < 10 μm) in an aqueous solution of several solutes (including sugars, acids, protein-like material, and caffeine) containing cell wall fragments of coffee solids measuring 2–5 μm [4]. Extraction results in a polyphasic colloidal system. A foam layer of small bubbles is formed on top of the aqueous solution, along with dispersed fine particles and microscopic oil droplets [5]. Therefore, coffee foam can be defined as a coarse biphasic system, made up of the liquid phase of the beverage, and small, spherical gas globules, each of which is surrounded by a *lamella* (a liquid film) that hosts biopolymers and natural surfactants [6]. In coffee-based beverages, the presence of persistent foam is considered an important quality attribute. In general, coffee specialists expect foam (*crema*) to fill at least 10% of the volume; it must survive at least two minutes before breaking up to reveal the underlying liquid phase. During its short lifetime, the *crema*’s structure and properties change considerably [7]. After formation, bubbles transform into dry foam on aging. Its disappearance is mainly attributed to drainage, characterized by the downward flow of trapped water towards the liquid phase under the influence of gravity, leaving only surfactant molecules to bear the stress of the lamella structure [8].

Recently, a new espresso brewing method, *Caffè Firenze* (EU Patent 06 023 798.9; US 2010/0034942 A1) has been developed. This method uses a sealed extraction chamber in which water and air are at higher pressures than other extraction methods, resulting in a pronounced difference in foam characteristics. The machine has been optimized following tests of the effects of different brewing settings (i.e., pressure and temperature) on the physical and chemical parameters of the resulting coffee [9]. Water temperature and pressure are recognized as being particularly important for the quality of traditional espresso [10]. Other studies have revealed the importance of overpressure during the extraction of specific compounds [11,12].

It is substantially different to the usual brewing method currently in commercial use: flow is the result of the pressure difference between the interior and the exterior of the chamber (rather than pressurized hot water provided by a motor-driven pump); extraction is partially static; pressure is higher than in the traditional method, and the temperature of the extraction chamber can be controlled [9]. Until now, air has been used to produce espresso. Traditional brewing machines are equipped with an electric compressor that feeds pressurized air into the extraction chamber.

Given the novel features of the extraction method, the aim of this research is to evaluate the effect of different food-grade gases, at a constant pressure of 20 bar, on foam quality/structure and the liquid. Several food-grade gases are used in industry as additives, either to support or in contact with ingredients. Their use can limit losses resulting from inefficient agricultural processes or other technological limitations encountered during storage, transportation, processing, cooling, or packaging [13]. Our choice of gases was evaluated from current knowledge about food preparation and conservation, taking into account the foaming potential of the gas. Five food-grade gases were tested (argon, nitrogen, nitrous oxide, and carbon dioxide), together with one mixture (carbon dioxide/nitrogen at a ratio of 70:30) and the standard configuration with air. All tests were run on the same coffee in order to accentuate any differences between gases and exclude any differences attributable to the type of coffee.

## 2. Materials and Methods

### 2.1. Experimental Design

The experiment was designed to highlight differences between the selected gases in terms of the physicochemical characteristics of foam and the liquid part of the beverage. While the chemical-physical characteristics of the liquid part of espresso coffee have been extensively studied, and standardized methods are described in the scientific literature, the physical properties of foam have received less attention. Consequently, we had to develop a repeatable, standardized measurement protocol for some parameters. Five replicates were run for each parameter and for each gas.

### 2.2. Coffee Samples and the Caffè Firenze Extraction Device

The same batch of 100% Arabica coffee (Illycaffè S.p.A, Trieste, Italy) was used for all extractions. Each packet of beans (250 g) was opened immediately before brewing to avoid oxidative damage. A professional grinder (EK43 Mahlkönig AG, Bachenbülach, Switzerland) was used to obtain a fine grind. All samples were prepared using the same commercial brand of mineral water (Via Nazionale, 2 Cepina Valdisotto 23020 Sondrio, Levissima, Italy). Coffee samples were collected according to the procedure reported in Angeloni et al. 2019 [1]. The *Caffè Firenze* extraction device is reported in Masella et al. (2015) [9] and consists, in brief, of the following parts:

− A sealed extraction chamber where coffee extraction takes place under high pressure (15 bar) and at a controlled temperature (80 °C).
− A system to feed pressurized gas into the extraction chamber.
− An electric pump to feed pressurized water (up to 20 bar) into the extraction chamber.
− An electric boiler to heat the water used for brewing.
− An electric resistance to heat the extraction chamber.
− A portafilter where the ground coffee is tamped; was equipped with a manual valve to allow the extraction chamber to be closed.
− Pressure and temperature transducers inside the extraction chamber.

### 2.3. Selection of Food-Grade Gases

Food-grade gases, either individually or in combination, are widely used in many different parts of the food industry and have very different properties. For our study, we selected five gases:

Carbon dioxide (CO_2_) is an ingredient in carbonated drinks and other beverages, such as juices. It is easily soluble in fat and water. In soft drinks, carbonation produces an appealing mouthfeel that is often described as ‘tingling’, and neurophysiological data indicate that CO_2_ stimulates both gustatory and trigeminal fibers [14,15].

Nitrogen (N_2_) is used in inert storage tanks and bottles in order to minimize oxidative damage due to aerobic bacteria. The principal mechanism is the replacement of O_2_ in the headspace [16]. Unlike CO_2_, which also has bacteriostatic properties and is highly soluble, N_2_ is virtually insoluble in water and fat and is used in modified atmosphere packaging as a filler gas.

Nitrous oxide (N_2_O) is an inert gas that is more fat-soluble than other gases and has water-soluble characteristics that are similar to CO_2_ [17]. This property is exploited in chocolate-making to improve sensory properties. N_2_O is also used to gasify the mixture in a coffee foamer before spray drying, and dried particles are used to produce the stable foaming creamer used in cappuccino. As it is more fat-soluble, it is suitable for whipping, and the overrun of whipped cream has been found to be better than normally-aerated cream [18].

The mixture of CO_2_ and N_2_ (70:30) is normally used for foam formation during brewing. Using N_2_ to replace some of the CO_2_ prevents the formation of carbonic acid, which is decisive for the final taste of the drink.

Argon (Ar) has no narcotic effect and is generally recognized as safe. It is preferred to other noble gases when the aim is to produce a foamy and creamy beverage. Ar is soluble in both lipid and water parts of the beverage and is mainly used for bottling wine in order to prevent oxidation and maintain quality [18].

Air consists of a mixture of gases: N_2_ (78.09%), O_2_ (20.95%), Ar (0.93%), CO_2_ (0.03%), and traces of neon, helium, methane, krypton, hydrogen, xenon, and ozone. Aeration plays an important role in the physical (textural) performance of some products, especially ice cream. In the latter case, the air is a key ingredient, and bubbles of air play a significant role in its sensory attributes, physical properties, and stability.

### 2.4. Foam Index Persistence and Consistency

The capacity of the cylindrical sampling vessel was 50 mL, with 42 mm external diameter, 60 mm high.

The foam index is defined as the ratio between foam and liquid (%), which was measured twice: 30 s and 2 min after extraction. Persistence is defined as the time (in minutes) before the foam breaks up, leaving an uncovered black spot on the surface of the beverage [19].

Foam consistency was measured with the sugar test, following the method described by Severini and co-author, [20] with minor modifications. Specifically, 1 g of granulated sugar was evenly scattered on the foam phase of extracted espresso coffee in the cylinder. In order to standardize the procedure, sugar was poured through a funnel positioned at a height of 10 cm. The retention time of the granulated sugar on the foam phase was measured by visual inspection, using a stopwatch. Data are presented as the average ± standard deviation of six independent extractions.

### 2.5. Rheological Measurements

The rheological response of samples was determined by the acquisition of flow curves. Measurements were performed using the Physica Rheolab MC1 (Paar Physica, Anton-Paar-Straße 20, Graz, Austria) rotational rheometer and a connected PC. The following parameters were set:

- The rotational speed of the cylinder: 300 rpm.
- The data acquisition time: 60 s.

The typical graduated cylinder (75 mL volume, 25 mm diameter) applied to semi-solid samples was used for extraction. Analyses were performed after 1 min, with samples placed in the same position for each measurement. The rheometer determines the correct torque required to ensure the constant rotational velocity of the cylinder.

### 2.6. Color

Foam images were acquired using a digital microscope (RS PRO USB digital Microscope, RS Components S.r.l.- Viale T. Edison 110, Edificio C, Sesto San Giovanni Milano, Italy) with a resolution of 10 × 300, 30 fps, 5 M pixel, mounted on a testing device designed to maintain a constant distance to the observer. Image J^®^ is completely free software, which is widely used in the field of microscopy.

The fine adjustments in the camera settings (focus distance, zoom, and white balance) were defined from preliminary experiments and maintained constant for all images, seeking to standardize the characteristics for image acquisition. For each sample, six images were acquired inside a chamber to reduce the influence of environmental conditions.

The images were obtained in the RGB color model, where information on the intensity of the red (R), green (G), and blue (B) colors is captured.

### 2.7. Volatile Organic Compounds

A photoionization detector is typically used to measure volatile organic compounds (VOCs) at low concentrations. The technology is based on ionization: ultraviolet light emitted by the sensor ionizes the gas target, and the voltage that is generated is amplified and measured. Data were recovered with Vernier LabQuest 2 software. The sensor was placed in the headspace of espresso samples for 900 s, and all samples were placed in the same position for each measurement.

### 2.8. Total Dissolved Solids, Caffeine and Chlorogenic Acids

Total dissolved solids (TDS) were measured using a refractometer (VST LAB Coffee III Refractometer, VST inc, Ann Arbor, MI, USA).

Coffee samples were centrifuged at 12,074× *g* for 5 min and diluted 1:10 with water before high-performance liquid chromatography (HPLC) analysis. HPLC was carried out using an Agilent HP 1100 system equipped with an autosampler, column heater module, and quaternary pump, coupled to a diode array detector (DAD) from Agilent Technologies (Palo Alto, Santa Clara, CA, USA). A 150 mm × 3 mm i.d., 2.7 μm Poroshell 120, ECC18 column (Agilent Technologies, Santa Clara, CA, USA) was used, equipped with a precolumn of the same phase, and maintained at room temperature. The analysis conditions were the same reported in detail in Angeloni et al., 2019 [21]; the elution method was performed at a flow rate of 0.4 mL/min using water at pH 3.2 by formic acid and acetonitrile, applying a multistep linear solvent gradient.

Chromatograms were registered at 278 and 330 nm for caffeine and chlorogenic acids (CGAs), respectively. Caffeine and CGAs were identified by comparing their retention times and ultra-violet–visible light (UV–Vis) spectra to those of the respective standard, when it was possible, or with our previous data.

CGAs were evaluated using a five-point CGA (99% purity) calibration curve (Extrasynthèse, Genay, France) at 330 nm (0–1.776 μg; r^2^ = 0.9991) and caffeine content was determined using a six-point calibration curve from Extrasynthèse (95% purity) at 278 nm (0–0.632 μg; r^2^ = 0.9994).

### 2.9. Statistical Analyses

Conventional analysis of variance (ANOVA) was applied to evaluate the effect of the six gases on the selected compounds. Where the univariate F-test was significant at the *p* < 0.05 levela and multiple paired means were checked for significance using the Tukey Honest Significance Difference (HSD) post hoc test (*p* < 0.05). A principal component analysis was applied to identify patterns and structures in the analyzed data. All statistical analyses were performed using R software (version 3.4.0 for Windows).

## 3. Results and Discussion

This section reports results for physical and chemical measurements relating to espresso made with different gases. The first part characterizes the extracted cream, in particular, the foam structure (volume, persistence and consistency, torque, VOCs, and color). The second part characterizes the liquid part of the extracted samples (TDS, caffeine, and CGAs).

### 3.1. Foam Structure

Table 1 reports results related to foam structure. A conventional ANOVA was applied to each parameter, except persistence. Significant differences were found for all tested parameters (*p* < 0.05) with respect to the gas used.

Persistence was evaluated as the time, in minutes, needed for the foam to dissolve. In the cases of N_2_O and CO_2_, this took under 90 s, while for air, Ar, and N_2_, it took over 8 h. Our results for air confirm previous research [9], while other gases have not been studied before. It is interesting to compare these results to other parameters, such as the foam index (see next section).

Measurements of the foam index after 30 s found a negative linear correlation (R = −0.8) with persistence. Persistence was highest when the foam index was lowest. This effect was attributed to the high foaming power of N_2_, N_2_O, and CO_2_, which are widely used to produce bubbles in food preparation.

In addition, CO_2_ is released during grinding. Illy and Navarini [8] recommend a maximum delay of 30 min after grinding to avoid CO_2_ loss. In our experiment, all ground bean samples were tested immediately. The quick-release of CO_2_ during brewing is particularly important in expresso making; for the formation of the foam layer. The thermal gradient of the *crema* is also relevant in interpreting the inverse correlation between the foam index and persistence. CO_2_ has low solubility in hot water, and this can destabilize the foam that is formed [8].

Nunes and co-authors [22] showed that espresso foamability and foam stability decrease with increasing water content in roasted ground beans, increase with increasing compression in the filter holder, and increase with increasing quantities of beans. All of our experimental trials were run under the same operating conditions, with the same beans. Only the gases were different; thus any differences in the foam index were attributable to the gas used.

The foam index was also measured after 2 min, to reflect real consumption conditions. The results were very different from the index measured after 30 s. For the two mixes of gas and CO_2_, the foam phase had totally dissolved in the liquid phase. This is particularly interesting for the optimization of the brewing machine, as a key characteristic of the system is the presence of a high level of foam, which has a significant influence on the coffee’s aesthetic appeal. The highest values were obtained for air, N_2_, and Ar. Moreover, these levels were higher with respect to traditional espresso. Illy and Viani [7] report that *crema* should represent at least 10% of the volume of espresso; the *Caffè Firenze* method resulted in a higher percentage. It should be noted that different trends were observed for foam indices measured at 30 s and 2 min. In particular, a positive correlation was observed between the 2-min index and persistence (R = 0.947), which provides an insight into the foam structure, notably its bubbles.

Consistency was determined by measuring the length of time scattered sugar remained on the foam phase. Here, trends were similar to persistence. Times (in seconds) was longest for extraction with air and Ar and shortest for CO_2_, the mixture of CO_2_ and N_2_, and N_2_O. The latter measurements were observed 1 min after extraction ended, and the sugar disappeared quickly along with the foam (as described above).

In general, for the other gases, the presence of foam increased the sugar retention time. For N_2_, the relationship between persistence and retention time was indirect. Although shorter times were recorded for Ar and air, measurements were more stable (lower standard deviation). Our findings can be compared to Severini and co-authors [20], who reported the foam consistency of espresso as 0.5–10 s. The latter results are much lower than our observations for air and Ar but similar to those for N_2_.

**Figure 1:**
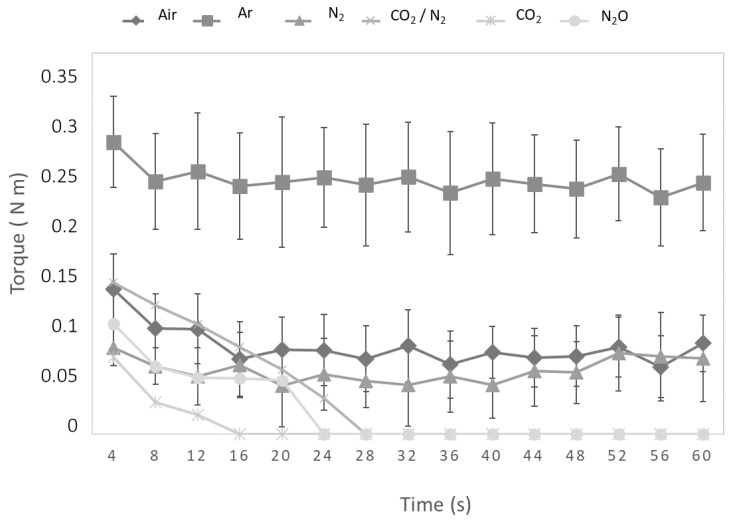
Torque measurements (N m) as a function of time(s) for the six gases.

The effect of the extraction gas on espresso coffee foam was measured as flow curves, representing torque as a function of time. The torque index is linked to foam consistency. Table 1 reports averages for each gas, while curves are shown in Figure 1.

The ANOVA revealed significant differences between gases (*p* ≤ 0.05); in particular, samples could be divided into two groups. The first comprised Ar, air, and N_2_; here, the foam persisted for the longest time. The other comprised the CO_2_/N_2_ mixture, CO_2_ and N_2_O; here, the foam disappeared so quickly that the measurement could not be completed. Figure 1 shows that, for all samples, the curve initially falls, as it is necessary to supply energy to break the structure. This is followed by a constant trend for air, Ar, and N_2_, which is typical of emulsions; as drops of liquid or gas form, bubbles are forced to stretch to facilitate the flow. Although similar results were observed for all samples, values were significantly higher for Ar than air and N_2_. For CO_2_ and N_2_O, complete data acquisition was not possible due to the collapse of the foam a few seconds after extraction.

**Figure 2:**
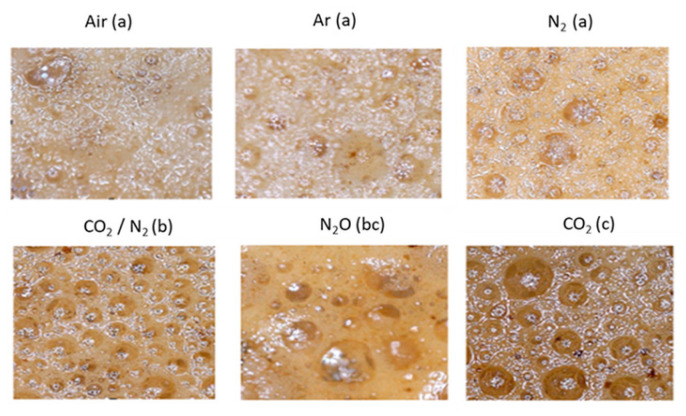
Images of *Caffè Firenze* espresso foam obtained with the six gases. Different letters indicate statistically significant differences (*p* < 0.05 Tukey’s Test).

Images of espresso samples were processed using ImageJ software in order to evaluate differences in foam color for the six gases. The ANOVA revealed significant differences for average red-green-blue (RGB) color coordinates (*p* ≤ 0.05). No significant differences were found between air, Ar, and N_2_ samples (Figure 2, top row), but these samples were different from the other three (N_2_O, CO_2_/N_2_, and CO_2_).

In particular, the CO_2_ sample was especially brown. TDS measurements suggest that this more intense color could be due to the high percentage of compounds dissolved in coffee extracted with the latter gases [23].

Color images made it possible to evaluate the bubble diameter, which appeared to be different. Bubbles in samples produced with air and Ar appeared to be smaller than other samples, while there were fewer larger bubbles compared to samples produced with mixed gases and CO_2,_ for which we have measured a diameter also ten times higher than the others. Foams, in general, are characterized by an irreversible reduction in the total number of bubbles and volume until their total collapse. In our experiment, each gas is soluble in each liquid (although to a different extent, depending on the case), and the foam evolves due to the diffusion of the gas phase through the film [24]. Both CO_2_ and N_2_O are highly soluble in water, and larger bubble size is associated with this property. An increase in the size of some bubbles is at the expense of others; globally, an increase in average bubble diameter is consistent with a decrease in their number. On the other hand, both N_2_ and Ar are much less soluble in water [25]. This process, called coarsening (enlargement), significantly affects the life of the foam.

**Figure 3:**
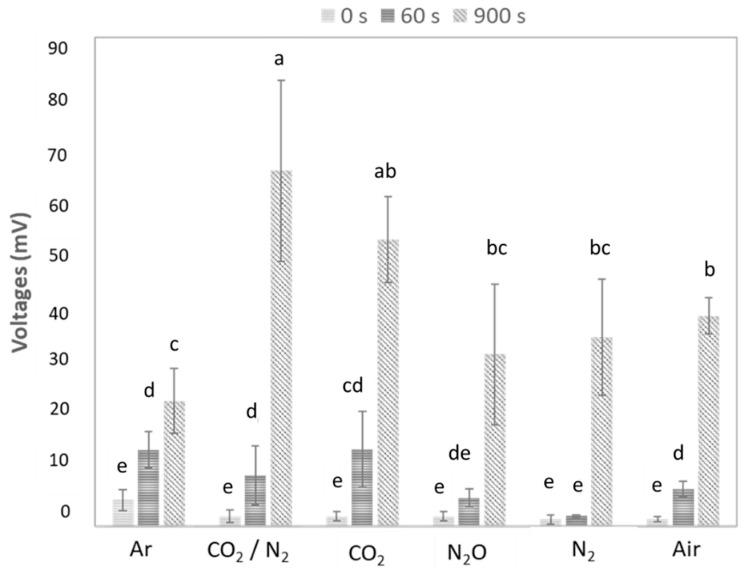
Summary of all pairwise comparisons for gas in the function of time (Tukey Honest Significance Difference (HSD)). Mean and standard deviation of voltages (mV). Different letters (a, b, c, d and e) indicate significant differences (*p* < 0.05).

VOCs were measured in the headspace of each sample. The aim was to evaluate if the foam acted as a barrier to their detection. Table 1 reports the mean for each gas, while Figure 3 shows the mean for each gas at three points in time: at the start of the analysis, after 60 s, and at the end of the measurement.

The ANOVA found no significant differences between gases for values registered immediately (0 s). As noted above, the analysis was repeated 1 min after sample preparation. Significant differences were observed between measurements recorded at 0 and 60 s for all gases except N_2_ and N_2_O. This was probably due to the progressive dissolution of the foam. There was an interesting increase in percentages within individual gases over time. Increases were lowest for N_2_ and N_2_O (50% and 70%, respectively), while an increase of up to six times was observed for other gases, for example, CO_2_.

At the final measurement time (900 s), significant differences were registered for all samples compared to the other measurements. Overall, voltages (mV) for all gases were highest with respect to other times. Among the gases, the highest values were registered for CO_2_ and CO_2_/N_2_. Here, at the end of the measurement time, the foam was either not present or was present and characterized by large bubbles. Finally, the same voltage was recorded for N_2_, air, and N_2_O, but values were lower for Ar.

We also analyzed variation in voltage. Variation was lowest for Ar samples, compared to the other gases—in the latter case, the increment related to the first measure was higher. It seems that Ar is more efficient in preventing the exit of VOCs than the other gases. This characteristic may be due to foam properties highlighted by rheological data (Figure 1). Not only does it persist over time, but also the dimensions and structure of its bubbles seemed to change the ability to release VOCs.

### 3.2. Analysis of Components in Solution

The ANOVA revealed significant differences between TDS in coffees produced with different gases (*p* ≤ 0.05). As shown in Table 2, TDS were highest for samples produced with N_2_O (4.76 ± 0.4%) and CO_2_ (4.21 ± 0.6%).

Results for air were similar to the other gases, and values were consistent with a previous study [1]. TDS values provide information about the percentage of dissolved compounds, and, in our experiment, they were consistent with the analysis of foam color.

The ANOVA revealed significant differences (*p* ≤ 0.05) in caffeine content (mg/mL) for the six gases. Values were highest for samples produced with CO_2_ (2.33 ± 0.13 mg/mL) and N_2_O (2.57 ± 0.11 mg/mL), and no significant difference was found between these two gases. Values were lowest in samples produced with N_2_ (1.32 ± 0.03 mg/mL), Ar (1.37 ± 0.09 mg/mL), and air (1.46 ± 0.04 mg/mL), while no significant differences were found between these three gases. Several studies have indicated that caffeine content ranges from 2.4 to 4.5 mg/mL for a traditional espresso [1,26]. Caffeine is moderately soluble in water at a room temperature of 20 °C (1.46 mg/mL), it increases at 80 °C (180 mg/mL), and becomes very soluble at 100 °C (670 mg/mL) [27]. The lower caffeine concentration in *Caffè Firenze* coffee could be due to the design of the chamber—the coffee panel is in direct contact with water at 80 °C [9], which is lower than in a classic espresso. The sample produced with air had caffeine content similar to samples produced with Ar and N_2_, and values were similar to those reported in a previous study [1]. However, concentrations in samples produced with CO_2_ and N_2_O were comparable to levels in standard espresso. Like the TDS measure, this finding shows the positive influence of CO_2_ and N_2_O on the capacity to extract caffeine.

Similar trends were found for CGA concentrations. Six caffeoylquinic acids, a feruloylquinic acid, a p-coumaroylquinic acid, four lactones of caffeoylquinic acid, and three dicaffeoylquinic acids were identified. Chlorogenic acid (5-Ocaffeoylquinic acid, 5-CQA) was most abundant, followed by its 3- and 4-CQA isomers, confirming a previous study [28]. The highest values were observed for samples produced using CO_2_ and N_2_O, while the lowest values were observed for samples extracted using Ar, air, and N_2_. In a previous study [1], the authors found higher total CGA content (about 4.43 mg/mL). This discrepancy could be due to the variety, origin, and, above all, the degree of roasting of beans, given their sensitivity to heat. On the other hand, caffeine content was similar in the two studies. It should be noted that caffeine is a thermostable molecule that only undergoes negligible losses during roasting.

### 3.3. Correlations between Parameters and Samples

**Figure 4:**
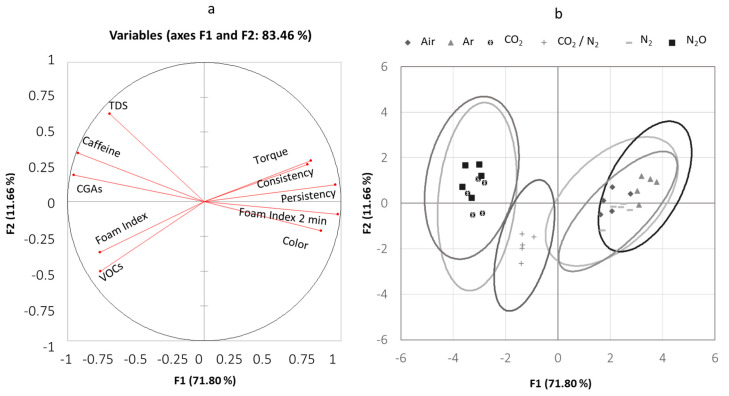
The first panel (**a**) represents the correlation between a variable and a principal component; the second panel, (**b**), represents the dispersion of factorial scores on the first and second axes.

A principal component analysis summarized differences related to the physical and chemical parameters of foam and liquids for the six gases. This confirmed that 83.5% of the total variance in the original dataset was explained by the first (71.80%) and second (11.66%) principal components, and these figures are a useful approximation of the relationships between variables (Figure 4a).

The angle between vectors is an approximation of the correlation between variables. A small angle indicates variables that are positively correlated, an angle of 90° indicates that they are not correlated, and an angle close to 180° indicates a negative correlation; see, for example, Color–Caffeine and CGA concentration, or CGA concentration and Foam Index after 2 min. The length of the line and its closeness to the circle indicates how well the variable is represented in the plot.

Multivariate analysis identified three groups of gases (Figure 4b).

The samples produced with Air, N_2_, and Ar were placed on the right side of the plot, while the CO_2_ and N_2_O samples on the left side with also the third gas that comprised samples extracted with a mixture of CO_2_/N_2_. Hence, the PCA analysis was of particular interest since it allowed to identify the variables that produced the differences between the gases adopted to produced coffee. The samples placed in the right side of the plot were characterized by higher values of Torque, Color, Consistency, Persistency, and Foam index after 2 min. The opposite result was obtained for the samples placed on the left side of the plot. For these gases, the highest values were found for TDS, Caffeine and CGAs, Foam Index, and VOCs.

The confidence ellipses of the samples produced with a mixture of CO_2_/N_2_ intersected with the other two groups. As this gas is a mix of the other two gases present in different groups, the intermediate trend is unsurprising.

## 4. Conclusions

Earlier work has observed that different gases can significantly influence the chemical-physical characteristics of the drink obtained and that the choice of gas is fundamental in diversifying production. Our results highlight the potential effect of six gases on both foam quality/structure and liquid coffee. Our measurements and methods made it possible to evaluate foam quality and the influence of different gases on both foam and liquid. If the aim is to produce coffee with more persistent foam and compact structure, which, however, acts as a barrier to VOCs, gases such as N_2_, air, and Ar should be preferred. If the aim is to increase concentrations of bioactive compounds, CO_2_ or a mix of gases should be selected. A mix consisting of CO_2_ and N_2_ often resulted in coffee with intermediate characteristics in terms of foam persistence and caffeine and CGA concentrations. This is likely to be due to the composition of the mix, which is a compromise between CO_2_ and N_2_ properties.

Future attempts to optimize the process could focus on the influence of the gas used for extraction on the sensory properties of coffee and the fraction of aromatic compounds. Furthermore, future evaluations should consider different coffee varieties. In this study, we have selected only one variety of coffee to identify the effect of the gases used. Probably it will be possible to observe the same trend with other varieties of coffee, with some differences for the compounds in solution, for example, for Caffeine and CGAs concentrations, strictly depending on the variety of coffee.

## Figures and Tables

**Table 1:** Mean and standard deviation of physical and chemical parameters. Different letters (a, b, c, and d) indicate statistically significant differences between different gases relative to the parameter measured. (*p* < 0.05 Tukey’s Test). Nd = not detected. VOCs: volatile organic compounds.

Gas	Air	Ar	CO_2_	CO_2_/N_2_	N_2_	N_2_O
Foam Persistence (min)	>480	>480	<2	15–18	>480	<2
Foam Index (%) after 30 s	60.40	50.20	76.10	88.80	40.20	78.80
	15.44	1.10	11.57	6.42	13.39	6.43
	b	c	ab	a	c	ab
Foam Index (%) after 2 m	32.20	34.00	Nd	13.30	28.20	Nd
	1.48	1.58		1.86	1.64	
	ab	a	d	c	b	d
Foam Consistency (s)	22.42	23.28	2.45	2.72	6.71	2.64
	6.71	5.08	0.21	0.38	0.67	0.09
	a	a	c	c	b	c
Torque (N m)	0.08	0.21	0.02	0.05	0.08	0.04
	0.03	0.05	0.00	0.03	0.01	0.02
	b	a	c	bc	b	bc
VOCs (mV)	28.00	17.30	43.32	48.77	20.60	36.79
	3.15	3.08	7.21	10.02	4.10	9.21
	bc	c	a	a	c	ab

**Table 2:** Mean and standard deviation of physical-chemical parameters measured in solution. Different letters (a, b, and c) indicate statistically significant differences (*p* < 0.05 Tukey’s Test).

GAS	Air	Ar	CO_2_	CO_2_/N_2_	N_2_	N_2_O
TDS (%)	3.36	2.98	4.21	2.77	2.62	4.76
	0.31	0.15	0.60	0.36	0.26	0.43
	b	b	a	b	b	a
Caffeine (mg/mL)	1.46	1.37	2.33	1.69	1.32	2.57
	0.04	0.09	0.13	0.07	0.03	0.11
	c	c	a	b	c	a
CGAs (mg/mL)	2.12	1.95	3.51	2.94	2.03	3.99
	0.11	0.15	0.35	0.21	0.04	0.24
	c	c	a	b	c	a

TDS: Total dissolved solids; CGA: chlorogenic acids.
